# Effect of air polishing on surface roughness in implant abutments

**DOI:** 10.6026/9732063002001667

**Published:** 2024-11-05

**Authors:** Neha Sah, Badr Bamusa, Nidhi Mehrotra, Asutosh Pradhan, Supriya Mishra, Srishty Goyal

**Affiliations:** 1 Department of Dentistry, Maa Vindhyavasini Autonomous State Medical College, Mirzapur, India; 2 Department of Preventive Dentistry, Riyadh Elm University, Riyadh, Saudi Arabia; 3 Department of Periodontology, Seema Dental College and Hospital, Rishikesh, Uttarakhand, India; 4 Master of Public Health, Asian Institute of Public Health, Bhubaneswar, Odisha, India; 5 Department of Periodontology, Government Dental College, Raipur, Chhattisgarh, India; 6 Department of Periodontology and Oral Implantology, Kalinga Institute of Dental Sciences, KIIT-DU, Bhubaneswar, Odisha, India

**Keywords:** Air polishing, implant abutment, sodium bicarbonate, erythritol powder

## Abstract

This *in vitro* investigation attempts to assess how air polishing with various abrasive powders affects implant abutment surface roughness. Thirty titanium implant abutments, split into three groups of ten each, were used in this *in vitro* investigation. Powdered glycine was given to Group A, powdered sodium bicarbonate to Group B and powdered erythritol to Group C. For 20 seconds, all abutments were air polished at a pressure of 60 psi and a nozzle distance of 5 mm. A profilometer assessed the surface roughness (Ra values) before and after treatment. Implant abutments' surface roughness changed very little after air polishing with erythritol and glycine powders. Therefore, implant care is possible. Sodium bicarbonate powder, on the other hand, dramatically increased surface roughness, which would raise the possibility of biofilm formation. Consequently, it is advised to regularly clean implant abutments using glycine and erythritol.

## Background:

The maintenance of dental implants is crucial for long-term success and a key factor in implant longevity is preventing the accumulation of biofilm on the abutment surfaces [1]. Biofilm build-up can lead to peri-implant diseases, including peri-implant microsites and peri-implantitis, which can compromise the stability and function of the implant [2]. Traditional methods of cleaning implant surfaces, such as scaling and polishing, may cause surface alterations that increase roughness, thereby enhancing biofilm adhesion [3]. Air polishing has emerged as a less invasive alternative that uses fine abrasive powders and pressurised air to remove biofilm without causing significant damage to the implant surface [4]. Different types of abrasive powders are used in air polishing, including glycine, sodium bicarbonate and erythritol. Each powder has unique properties that may affect its interaction with the implant surface [5]. Glycine and erythritol are fine, low-abrasive powders that have been shown to effectively remove biofilm without significantly altering the implant surface roughness [6]. In contrast, sodium bicarbonate, a coarser abrasive, may cause surface alterations that increase roughness and potentially enhance bacterial adhesion [7]. Maintaining the smoothness of the implant abutment surface is important, as increased roughness can facilitate bacterial colonisation and biofilm formation, leading to a higher risk of peri-implant complications [8]. Previous studies have investigated the effects of air polishing on natural teeth and restorative materials, but limited research exists on its impact on implant abutment surfaces [9]. This study aims to evaluate and compare the effects of air polishing using glycine, sodium bicarbonate and erythritol powders on the surface roughness of implant abutments *in vitro*.

## Methods and Materials:

This *in vitro* experimental investigation aimed to assess how air polishing with various abrasive powders affected the titanium implant abutments' surface roughness. Thirty titanium abutments were chosen and arbitrarily split into three groups of ten abutments each for the air polishing process using an abrasive powder of a certain kind. The extensive use of titanium implant abutments in dental Implantology led to their selection. Before the air polishing procedure started, the cylindrical abutments with a uniform surface finish were carefully washed with deionized water and allowed to dry. A commercially available air-polishing apparatus (Airflow® Prophylaxis Master, EMS and Switzerland) was used for the air-polishing treatment. The nozzle was mounted at a distance of 5 mm from the surface of the abutment and the device was adjusted to a constant air pressure of 60 psi. Air polishing was applied to each abutment in a circular motion for twenty seconds to guarantee that the whole surface received the same level of care. The following abrasive powders were utilized for the air polishing: ultra-low-abrasive erythritol powder (14 µm particle size), medium-abrasive sodium bicarbonate powder (65 µm particle size) for Group B, low-abrasive glycine powder (25µm particle size) for Group A. A profilometer (MitutoyoSurftest SJ-410, Japan) was used to assess the surface roughness both before and after the air polishing process. An average surface roughness (Ra) value was determined by measuring the surface roughness at three separate sites on each abutment after calibrating the profilometer before each usage. The abutments' initial surface roughness, or pre-polishing, varied between 0.6 and 0.8 µm. The surface roughness parameters were analysed statistically by calculating each group's mean and standard deviation. The three groups' variations in surface roughness were compared using a one-way analysis of variance (ANOVA). Less than 0.05 was the threshold for statistical significance. This investigation's results provide light on how various abrasive powders modify the surface properties of titanium implant abutments, which might have practical applications in the field of dental Implantology.

## Results:

A total of 30 titanium implant abutments were polished using three different abrasive powders. Surface roughness (Ra) values were measured before and after the air polishing procedure. The mean surface roughness values and standard deviations (SD) for each group are presented in Table 1. The pre-polishing Ra values for all groups were comparable, ranging from 0.71 µm to 0.74 µm. After air polishing, the glycine group (Group A) showed a reduction in surface roughness, with a mean post-polishing Ra of 0.62 µm, resulting in a mean decrease of 0.10 µm. The erythritol group (Group C) exhibited a similar reduction in roughness, with a mean post-polishing Ra of 0.61 µm and a mean decrease of 0.10 µm. In contrast, the sodium bicarbonate group (Group B) demonstrated an increase in surface roughness, with a post-polishing Ra of 0.85 µm, resulting in a mean increase of 0.11 µm. Statistical analysis using one-way ANOVA revealed a significant difference in surface roughness between Group B and Groups A and C (p < 0.05), indicating that sodium bicarbonate caused a significant increase in surface roughness compared to glycine and erythritol. However, no significant difference was observed between Group A (glycine) and Group C (erythritol) (p > 0.05) (Table 2).

## Discussion:

The current *in vitro* investigation aimed to assess how air polishing with three distinct abrasive powders-erythritol, sodium bicarbonate and glycine-affected the surface roughness of titanium implant abutments. Preventing biofilm buildup, which may lead to peri-implant illnesses such as peri-implant mucositis and peri-implantitis, requires maintaining a smooth implant abutment surface [1]. Prior research has shown that implant abutment surface roughness affects bacterial colonisation, with rougher surfaces more likely to develop biofilms [2, 3]. Our findings showed that whereas sodium bicarbonate significantly increased surface roughness, glycine and erythritol powders had no effect. This is in line with the results of Schwarz *et al.* [4], who found that sodium bicarbonate may considerably roughen titanium surfaces because of its greater particle size. In agreement with our results, Petersilka *et al.* [5] discovered that glycine powder air polishing maintained the surface integrity of implant abutments. The glycine and erythritol groups' reduced surface roughness may be ascribed to their tiny particle sizes, which facilitate efficient biofilm clearance without resulting in significant surface modifications [6]. Further supporting our findings, Müller *et al.* [7] discovered that erythritol and glycine were kinder on implant surfaces than sodium bicarbonate. Maintaining the smoothness of the surface is crucial for these groups since roughened surfaces may promote bacterial adherence and raise the risk of peri-implant infections [8, 9]. Conversely, upon polishing, the sodium bicarbonate group exhibited a notable rise in surface roughness, consistent with the findings of Sahm *et al.* [10]. It has been shown that the larger sodium bicarbonate particles abrade titanium surfaces, which might shorten the life of the implant by encouraging bacterial colonisation [11]. The results of Quirynen *et al.* [12], who showed that rougher surfaces promote faster and denser biofilm development, are consistent with this. Sodium bicarbonate and the other two powders (glycine and erythritol) differed significantly in this study's statistical analysis, suggesting that sodium bicarbonate may not be the best option for implant care when surface integrity is a top concern [13]. Glycine is suggested by Jepsen *et al.* [14] for peri-implant care, underscoring the need to choose polishing powders that maintain surface smoothness. In addition to the arsenal of air-polishing powders, erythritol has shown encouraging outcomes in preserving surface integrity and efficiently eliminating biofilm [15]. Our study's main consequence is that physicians must carefully choose air-polishing powders depending on how they affect the properties of the implant surface. Because they do not affect surface roughness, glycine and erythritol are appropriate for regular implant care, as shown by this study and other studies [16]. On the other hand, because of its abrasive properties, sodium bicarbonate should be used with care as it may increase surface roughness and the risk of peri-implant disorders [7]. Our results align with previous research emphasising the importance of air polishing in preserving implant health without generating surface degradation [8]. It is crucial to remember that this study was carried out *in vitro* and more clinical research is required to confirm the benefits of these polishing powders *in vivo* [9]. Moreover, characteristics unique to each patient and the length and pressure of air polishing might affect the outcome in a clinical context [10].

## Conclusion:

This study confirms that glycine and erythritol powders effectively maintain the smoothness of implant abutment surfaces, whereas sodium bicarbonate increases surface roughness and may enhance biofilm formation. Clinicians should prioritise the use of low-abrasive powders such as glycine and erythritol for implant maintenance to minimise the risk of peri-implant complications. Future research should focus on long-term clinical outcomes of different air polishing powders and their effects on peri-implant health.

## Figures and Tables

**Table 1:** Surface roughness (RA) values before and after air polishing

**Group**	**Abrasive powder**	**Pre-polishing ra (µm) mean ± sd**	**Post-polishing ra (µm) Mean ± sd**	**Difference in ra (µm) Mean ± sd**
Group A	Glycine	0.72 ± 0.03	0.62 ± 0.04	-0.10 ± 0.03
Group B	Sodium Bicarbonate	0.74 ± 0.05	0.85 ± 0.06	+0.11 ± 0.04
Group C	Erythritol	0.71 ± 0.02	0.61 ± 0.03	-0.10 ± 0.02

**Table 2:** Statistical analysis (ANOVA) of surface roughness changes among groups

**Comparison**	**p-Value**	**Significance**
Group A vs. Group B	0.01	Significant (p < 0.05)
Group A vs. Group C	0.78	Not Significant
Group B vs. Group C	0.01	Significant (p < 0.05)
